# Correction: Comparative study of PRPH2 D2 loop mutants reveals divergent disease mechanism in rods and cones

**DOI:** 10.1007/s00018-023-04929-y

**Published:** 2023-09-12

**Authors:** Larissa Ikelle, Mustafa Makia, Tylor Lewis, Ryan Crane, Mashal Kakakhel, Shannon M. Conley, James R. Birtley, Vadim Y. Arshavsky, Muayyad R. Al-Ubaidi, Muna I. Naash

**Affiliations:** 1 Department of Biomedical Engineering, University of Houston, 3517 Cullen Blvd. Room 2027, Houston, TX 77204-5060 USA; 2 Department of Ophthalmology, Duke University Medical Center, Durham, NC USA; 3 Department of Cell Biology, University of Oklahoma Health Sciences Center, Oklahoma City, OK 73104 USA; 4 Epsilogen Ltd, Hammersmith, London, W6 9RH UK

**Correction: Cellular and Molecular Life Sciences (2023) 80:214** 10.1007/s00018-023-04851-3

In the original published article, the ESM files was missed during acceptance and was unnoticed in the subsequent stages. The ESM as follows.

### Supplementary Information

Below is the link to the electronic supplementary material.

Supplementary file1 (DOCX 3459 kb)

Supplementary file2 (MP4 1635 kb)

Supplementary file3 (MP4 1570 kb)

Supplementary file4 (MP4 1549 kb)

Supplementary file5 (MP4 1662 kb)

